# Supplementary material for: Sturnidae sensu lato Mitogenomics: Novel Insights into Codon Aversion, Selection, and Phylogeny
Source: Animals (Basel). 2024 Sep 26;14(19):2777. doi: 10.3390/ani14192777 (PMC11475038; doi:10.3390/ani14192777)
Supplement: Supplementary file 1 [file animals-14-02777-s001.zip › animals-3166609-supplementary.pdf]

Supplementary files

Table S1. Primers used in amplifying the mitogenome of *Acridotheres cristatellus*.

Table S2. Primers used in amplifying the mitogenome of *Acridotheres tristis*.

Table S3. Primers used in amplifying the mitogenomes of *Gracupica nigricollis*.

Table S4. Primers used in amplifying the mitogenome of *Spodiopsar cineraceus*.

Table S5. Sampling and GenBank accession numbers for multi-gene analysis.

Table S6. Species of mitogenomes examined in this study.

Table S7. The ENC and GC3s of PCGs examined in this study.

Table S8. The best Bayesian evolutionary models of combined dataset.

Table S9. Determination of substitution saturation of PCGs for combined dataset.

Table S1. Primers used in amplifying the mitogenome of *Acridotheres cristatellus.*

No. of primer pair	Name	Sequences (5’-3’)	Size (bp)
Long-PCR amplification			
1	QPASMT1F	CCAACCTCCTAATTCTCACC	20
	KLPASMTR3	ACTCTTTGTTGATGGCTGCT	20
2	KLPASMTF4	GAGGTGAAAAGCCAATCGAGC	21
	KLPASMTR7	ATGGATAGGACGTAGTGGAA	20
3	KLPASMTF8	GAATGGACGTAGACACCCG	19
	KLPASMTR10	GTTTTGCCTGGGTAGTATG	19
4	KLPASMTF10	AGAACTAGGAGGACAATGAC	20
	KLPASMTR12	CTTTTGAGTAGAATCCTGCT	20
5	KLPASMTF12	AAAACCTTCTTACCTGCCGA	20
	KLPASMTR14	GGCTTACAAGACCAATG	17
Nested PCR amplification			
1	QPASMT1F	CCAACCTCCTAATTCTCACC	20
	KLPASMTR1	TTGTGGTTTTGGAGCTTGTACG	22
2	KLPASMTF2	ATCTCCAACTCCCAAAGCT	19
	QPASbgR2	AAGCTACAAGGACAGTGAGG	20
	QPASCF2	CCATAGTAGGGAGAAGGGTT	20
	KLPASMTR2	GGTATCTAATCCCAGTTTG	19
3	KLPASMTF3	CCCACGGGTATTCAGCAGT	19
	KLPASMTR3	ACTCTTTGTTGATGGCTGCT	20
4	KLPASMTF4	GAGGTGAAAAGCCAATCGAGC	21
	KLPASMTR4	GCTAGGGAGAGGATTTGAACC	21
5	KLPASMTF5	AGTCCTACGTGATCTGAGTT	20
	KLPASMTR5	GGCCCGATAGCTTGTTTAG	19
6	KLPASMTF6	GATAAAGTGAACATAGAGGT	20
	QPASMT6R	GGTTCGGGCATATAGCTGTT	20
7	KLPASMTF7	GCCTTCAAAGCCTTAAACAA	20
	KLPASMTR7	ATGGATAGGACGTAGTGGAA	20
8	KLPASMTF8	GAATGGACGTAGACACCCG	19
	QPASMT8R	GAGGCATACCATTAAGGAGG	20
9	QPASMTF9	CGGACAGTGCTCAGAAATCT	20
	KLPASMTR9	GCTTCTGTAATACTGTGGTG	20
10	KLPASMTF10	AGAACTAGGAGGACAATGAC	20
	KLPASMTR10	GTTTTGCCTGGGTAGTATG	19
11	KLPASMTF11	CCTCCTACAATGCTAAAAAT	20
	KLPASMTR11	CTTTCACTTGGATTTGCACC	20
12	KLPASMTF12	AAAACCTTCTTACCTGCCGA	20
	KLPASMTR12	CTTTTGAGTAGAATCCTGCT	20
13	KLPASMTF13	TCCTACACATCTCAACGCAC	20
	KLPASMTR13	GAAGGCAGTTGCTATGAGG	19
14	KLPASMTF14	CCCACACCATCAAACATCTC	20
	KLPASMTR14	GGCTTACAAGACCAATG	17

Table S2. Primers used in amplifying the mitogenome of *Acridotheres tristis.*

No. of primer pair	Name	Sequences (5'-3')	Size (bp)
Long-PCR amplification			
1	KLPASMTF2	ATCTCCAACTCCCAAAGCT	19
	KLPASMTR3	ACTCTTTGTTGATGGCTGCT	20
2	KLPASMTF4	GAGGTGAAAAGCCAATCGAGC	21
	KLPASMTR7	ATGGATAGGACGTAGTGGAA	20
3	KLPASMTF7	GCCTTCAAAGCCTTAAACAA	20
	KLPASMTR10	GTTTTGCCTGGGTAGTATG	19
4	KLPASMTF13	TCCTACACATCTCAACGCAC	20
	KLPASMTR1	TTGTGGTTTTGGAGCTTGTACG	22
Nested PCR amplification			
1	KLPASMTF14	CCCACACCATCAAACATCTC	20
	QPASMTR1	TTTGTGGTGCTGGAGCTTGT	20
2	KLPASMTF2	ATCTCCAACTCCCAAAGCT	19
	QPASCF2	CCATAGTAGGGAGAAGGGTT	20
	QPASCR2	CAGTGTGATTGAATAGTGGC	20
	KLPASMTR2	GGTATCTAATCCCAGTTTG	19
3	KLPASMTF3	CCCACGGGTATTCAGCAGT	19
	KLPASMTR3	ACTCTTTGTTGATGGCTGCT	20
4	KLPASMTF4	GAGGTGAAAAGCCAATCGAGC	21
	KLPASMTR4	GCTAGGGAGAGGATTTGAACC	21
5	KLPASMTF5	AGTCCTACGTGATCTGAGTT	20
	KLPASMTR5	GGCCCGATAGCTTGTTTAG	19
6	KLPASMTF6	GATAAAGTGAACATAGAGGT	20
	QPASMT6R	GGTTCGGGCATATAGCTGTT	20
7	KLPASMTF7	GCCTTCAAAGCCTTAAACAA	20
	KLPASMTR7	ATGGATAGGACGTAGTGGAA	20
8	KLPASMTF8	GAATGGACGTAGACACCCG	19
	QPASMT8R	GAGGCATACCATTAAGGAGG	20
9	QPASMTF9	CGGACAGTGCTCAGAAATCT	20
	KLPASMTR9	GCTTCTGTAATACTGTGGTG	20
10	KLPASMTF10	AGAACTAGGAGGACAATGAC	20
	KLPASMTR10	GTTTTGCCTGGGTAGTATG	19
11	KLPASMTF11	CCTCCTACAATGCTAAAAAT	20
	KLPASMTR11	CTTTCACTTGGATTTGCACC	20
12	KLPASMTF12	AAAACCTTCTTACCTGCCGA	20
	KLPASMTR12	CTTTTGAGTAGAATCCTGCT	20
13	KLPASMTF13	TCCTACACATCTCAACGCAC	20
	KLPASMTR13	GAAGGCAGTTGCTATGAGG	19

Table S3. Primers used in amplifying the mitogenomes of *Gracupica nigricollis.*

No. of primer pair	Name	Sequences (5'-3')	Size (bp)
Long-PCR amplification			
1	KLPASMTF12	AAAACCTTCTTACCTGCCGA	20
	QPASMTR1	TTTGTGGTGCTGGAGCTTGT	20
2	KLPASMTF2	ATCTCCAACTCCCAAAGCT	19
	KLPASMTR4	GCTAGGGAGAGGATTTGAACC	21
3	KLPASMTF5	AGTCCTACGTGATCTGAGTT	20
	KLPASMTR7	ATGGATAGGACGTAGTGGAA	20
4	KLPASMTF8	GAATGGACGTAGACACCCG	19
	KLPASMTR11	CTTTCACTTGGATTTGCACC	20
Nested PCR amplification			
1	QPASJBF1	CGACCCATCTCACAAATCCT	20
	QPASMTR1	TTTGTGGTGCTGGAGCTTGT	20
2	KLPASMTF2	ATCTCCAACTCCCAAAGCT	19
	QPASblnR2	AAGGATGTGACTGGAAGGGT	20
	QPASCF2	CCATAGTAGGGAGAAGGGTT	20
	PaStMT2F	TTACCCTTCCAGTCACATCC	20
	KLPASMTR2	GGTATCTAATCCCAGTTTG	19
3	KLPASMTF3	CCCACGGGTATTCAGCAGT	19
	KLPASMTR3	ACTCTTTGTTGATGGCTGCT	20
4	KLPASMTF4	GAGGTGAAAAGCCAATCGAGC	21
	KLPASMTR4	GCTAGGGAGAGGATTTGAACC	21
5	KLPASMTF5	AGTCCTACGTGATCTGAGTT	20
	KLPASMTR5	GGCCCGATAGCTTGTTTAG	19
6	KLPASMTF6	GATAAAGTGAACATAGAGGT	20
	QPASMT6R	GGTTCGGGCATATAGCTGTT	20
7	KLPASMTF7	GCCTTCAAAGCCTTAAACAA	20
	KLPASMTR7	ATGGATAGGACGTAGTGGAA	20
8	KLPASMTF8	GAATGGACGTAGACACCCG	19
	QPASMT8R	GAGGCATACCATTAAGGAGG	20
9	QPASMTF9	CGGACAGTGCTCAGAAATCT	20
	QPASBMTR9	CCTCTGTGATGCTGTGATG	19
10	KLPASMTF10	AGAACTAGGAGGACAATGAC	20
	KLPASMTR10	GTTTTGCCTGGGTAGTATG	19
11	KLPASMTF11	CCTCCTACAATGCTAAAAAT	20
	KLPASMTR11	CTTTCACTTGGATTTGCACC	20
12	KLPASMTF12	AAAACCTTCTTACCTGCCGA	20
	KLPASMTR12	CTTTTGAGTAGAATCCTGCT	20
13	KLPASMTF13	TCCTACACATCTCAACGCAC	20
	KLPASMTR13	GAAGGCAGTTGCTATGAGG	19
14	KLPASMTF14	CCCACACCATCAAACATCTC	20
	KLPASMTR14	GGCTTACAAGACCAATG	17

Table S4. Primers used in amplifying the mitogenome of *Spodiopsar cineraceus.*

No. of primer pair	Name	Sequences (5'-3')	Size (bp)
Long-PCR amplification			
1	QPASMT1F	CCAACCTCCTAATTCTCACC	20
	KLPASMTR3	ACTCTTTGTTGATGGCTGCT	20
2	KLPASMTF4	GAGGTGAAAAGCCAATCGAGC	21
	KLPASMTR7	ATGGATAGGACGTAGTGGAA	20
3	KLPASMTF8	GAATGGACGTAGACACCCG	19
	KLPASMTR11	CTTTCACTTGGATTTGCACC	20
4	KLPASMTF12	AAAACCTTCTTACCTGCCGA	20
	KLPASMTR1	TTGTGGTTTTGGAGCTTGTACG	22
Nested PCR amplification			
1	QPASMT1F	CCAACCTCCTAATTCTCACC	20
	KLPASMTR1	TTGTGGTTTTGGAGCTTGTACG	22
2	KLPASMTF2	ATCTCCAACTCCCAAAGCT	19
	PaStMT2R	GTGTGGTTGGAATTGGCAG	19
	PaStMT2F	TTACCCTTCCAGTCACATCC	20
	KLPASMTR2	GGTATCTAATCCCAGTTTG	19
3	KLPASMTF3	CCCACGGGTATTCAGCAGT	19
	KLPASMTR3	ACTCTTTGTTGATGGCTGCT	20
4	KLPASMTF4	GAGGTGAAAAGCCAATCGAGC	21
	KLPASMTR4	GCTAGGGAGAGGATTTGAACC	21
5	KLPASMTF5	AGTCCTACGTGATCTGAGTT	20
	KLPASMTR5	GGCCCGATAGCTTGTTTAG	19
6	KLPASMTF6	GATAAAGTGAACATAGAGGT	20
	QPASMT6R	GGTTCGGGCATATAGCTGTT	20
7	KLPASMTF7	GCCTTCAAAGCCTTAAACAA	20
	KLPASMTR7	ATGGATAGGACGTAGTGGAA	20
8	KLPASMTF8	GAATGGACGTAGACACCCG	19
	QPASMT8R	GAGGCATACCATTAAGGAGG	20
9	QPASMTF9	CGGACAGTGCTCAGAAATCT	20
	KLPASMTR9	GCTTCTGTAATACTGTGGTG	20
10	KLPASMTF10	AGAACTAGGAGGACAATGAC	20
	KLPASMTR10	GTTTTGCCTGGGTAGTATG	19
11	KLPASMTF11	CCTCCTACAATGCTAAAAAT	20
	KLPASMTR11	CTTTCACTTGGATTTGCACC	20
12	KLPASMTF12	AAAACCTTCTTACCTGCCGA	20
	KLPASMTR12	CTTTTGAGTAGAATCCTGCT	20
13	KLPASMTF13	TCCTACACATCTCAACGCAC	20
	KLPASMTR13	GAAGGCAGTTGCTATGAGG	19
14	KLPASMTF14	CCCACACCATCAAACATCTC	20
	KLPASMTR14	GGCTTACAAGACCAATG	17

Table S5. Sampling and GenBank accession numbers for multi-gene analysis.

Family	Species	MT-ATP6	MT-ATP8	MT-CO1	MT-CO2	MT-ND2	Fib5	Fib7	RDP1	TGFB2-4	ODC	GAPDH-11	myo	RAG-1
Buphagidae	*Buphagus africanus*	EF486773	EF486773	EF484214	EF484314	EF468188	EF468320	EF471840	EF472852	EF484111				
Buphagidae	*Buphagus erythrorynchus*	MN356312	MN356312	MN356312	MN356312	MN356312							DQ466819	AY307183
Mimidae	*Allenia fusca*	EF486779	EF486779	AF141004	EF484320	EF468195	JN799765	JN799731	JN799834	JN799799			AF140880	
Mimidae	*Cinclocerthia gutturalis*	AF140900	AF140900	JN805990	JN806024	JN799700	JN799767	JN799733	JN799836	JN799801			AF140875	
Mimidae	*Cinclocerthia ruficauda*	JN806059	JN806059	JN805991	JN806025	JN799699	JN799766	JN799732	JN799835	JN799800			AF140877	
Mimidae	*Dumetella carolinensis*	JN806061	JN806061	AF141002	EF484317	JN799662	JN799735	JN799702	JN799803	JN799769			AF140878	AY319981
Mimidae	*Margarops fuscatus*	EF486778	EF486778	EF484218	EF484319	EF468194	EF468325	EF471846	EF472858	EF484117			AF140879	AY307197
Mimidae	*Melanoptila glabrirostris*	JN806067	JN806067	JN805999	EF484322	JN799663	EF468328	JN799703	EF472861	JN799770			AF140881	
Mimidae	*Melanotis caerulescens*	JN806062	JN806062	AF141006	EF484318	JN799697	JN799764	JN799730	JN799833	JN799798			AF140882	AY307199
Mimidae	*Melanotis hypoleucus*	EF486783	EF486783	EF484223	EF484324	EF468199	EF468330	EF471851	EF472863	EF484122				
Mimidae	*Mimus dorsalis*	JN806063	JN806063	JN805995	JN806029	JN799677	JN799745	JN799713	JN799814	JN799779				
Mimidae	*Mimus gilvus*	JN806066	JN806066	JQ175387	EF484321	JN799670	JN799738	JN799706	JN799807	JN799772			AF140883	
Mimidae	*Mimus graysoni*	JN806089	JN806089	JN806021	JN806055	JN799678								
Mimidae	*Mimus gundlachii*	AF140975	AF140975	AF141008	EF484323	EF468198	EF468329	EF471850	EF472862	EF484121			AF140884	
Mimidae	*Mimus longicaudatus*	JN806068	JN806068	DQ083936	EF484325	JN799673	JN799741	JN799709	JN799810	JN799775				
Mimidae	*Mimus macdonaldi*	DQ087196	DQ087196	DQ083930		AY311566	JN799749	JN799717	JN799818	JN799783				
Mimidae	*Mimus melanotis*	DQ087197	DQ087197	DQ083931		AY311577	JN799750	JN799718	JN799819	JN799784				
Mimidae	*Mimus parvulus*	DQ087201	DQ087201	DQ083935		KF411093	JN799747	KF411120	JN799816	JN799781				

Family	Species	MT-ATP6	MT-ATP8	MT-CO1	MT-CO2	MT-ND2	Fib5	Fib7	RDP1	TGFB2-4	ODC	GAPDH-11	myo	RAG-1
Mimidae	*Mimus patagonicus*	JN806071	JN806071	DQ083939	EF484326	JN799675	JN799743	JN799711	JN799812	JN799777				AY057005
Mimidae	*Mimus polyglottos*	BK010995	BK010995	BK010995	BK010995	BK010995	EF468333	JN799704	JN799805	JN799771			AF140885	
Mimidae	*Mimus saturninus*	JN806073	JN806073	JN806005	JN806039	JN799674	JN799742	JN799710	JN799811	JN799776	EU680742	KF905609	AY228304	AY037852
Mimidae	*Mimus thenca*	EF486788	EF486788	MG263846	EF484329	AY311539	EF468335	EF471856	EF472868	EF484127				
Mimidae	*Mimus trifasciatus*	DQ087195	DQ087195	DQ083929		AY311551	JN799748	JN799716	JN799817	JN799782				
Mimidae	*Mimus triurus*	DQ087204	DQ087204	DQ083938	EF484330	AY311533	EF468336	EF471857	EF472869	EF484128				
Mimidae	*Oreoscoptes montanus*	DQ087207	DQ087207	DQ083941	EF484331	AY758197	EF468337	EF471858	EF472870	EF484129				AY307203
Mimidae	*Ramphocinclus brachyurus*	AF140986	AF140986	AF141010	EF484332	EF468207	EF468338	EF471859	EF472871	EF484130			AF140886	AY307209
Mimidae	*Toxostoma bendirei*	JN806076	JN806076	JN806008	JN806042	JN799681	JN799753	JN799722	JN799823	JN799789				
Mimidae	*Toxostoma cinereum*	EF486792	EF486792	EF484232	EF484333	EF468208	EF468339	EF471860	EF472872	EF484131				
Mimidae	*Toxostoma crissale*	JN806078	JN806078	JN806010	JN806044	KY460136	JN799757	JN799724	KY460266	JN799791	KY460441		KY460418	
Mimidae	*Toxostoma curvirostre*	JN806080	JN806080	JN806012	JN806046	JN799685								AY307213
Mimidae	*Toxostoma guttatum*	JN806090	JN806090	JN806022	JN806056	JN799690								
Mimidae	*Toxostoma lecontei*	JN806082	JN806082	JN806014	JN806048	KY460167	JN799759	JN799725	KY460265	JN799793	KY460440		KY460421	
Mimidae	*Toxostoma longirostre*	JN806084	JN806084	JN806016	JN806050	JN799689	JN799755	JN799721	JN799822	JN799787				
Mimidae	*Toxostoma redivivum*	MN356247	MN356247	MN356247	MN356247	MN356247	JN799763	JN799729	JN799832	JN799797	KY460422		KY460417	
Mimidae	*Toxostoma rufum*	DQ087208	DQ087208	JN806006	EF484334	JN799679	JN799751	JN799719	JN799820	JN799785				
Sturnidae	*Acridotheres albocinctus*				EU551980	EU403586					EU551905	EU551874	EU551855	
Sturnidae	*Acridotheres burmannicus*				EU552004	EU403588						EU551885	EU551863	
Sturnidae	*Acridotheres cinereus*				EU551983	EU403590						EU551875	EU551856	
Sturnidae	*Acridotheres cristatellus*	NC_015613	NC_015613	NC_015613	NC_015613	NC_015613	EF468303	EF471822	EF472836	EF484093	EU551907	EU551876	EU551857	

Family	Species	MT-ATP6	MT-ATP8	MT-CO1	MT-CO2	MT-ND2	Fib5	Fib7	RDP1	TGFB2-4	ODC	GAPDH-11	myo	RAG-1
Sturnidae	*Acridotheres fuscus*	EF486755	EF486755	EF484196	EF484296	EU551942	EF468304	EF471823	EF472837	EF484094	EU551908	EU551877	DQ466813	AY307180
Sturnidae	*Acridotheres ginginianus*	EF486756	EF486756	EU525243	EF484297	EU403591								
Sturnidae	*Acridotheres grandis*	EF486757	EF486757	EF484198	EF484298	EF468168	EF468305	EF471824	EF472838	EF484095				
Sturnidae	*Acridotheres javanicus*	EF486758	EF486758	EF484199	EF484299	EU403593	EF468306	EF471825	EF472839	EF484096	EU551910	EU551879	EU551859	
Sturnidae	*Acridotheres melanopterus*				EU551993	EU403594					EU551911	EU551880	EU551860	
Sturnidae	*Acridotheres tristis*	NC_015195	NC_015195	NC_015195	NC_015195	NC_015195	EF468307	EF471826	EF472840	EF484097	EU551912	EU551881	DQ466814	DQ466794
Sturnidae	*Agropsar philippensis*	EF486767	EF486767	EF484208	EU552015	EF468179	EF468314	EF471834	EF472846	EF484105	EU551923	EU551892	EU551868	
Sturnidae	*Agropsar sturninus*				EU552020	EU551970					EU551927	EU551896	EU551871	
Sturnidae	*Ampeliceps coronatus*	EF486737	EF486737	EF484178	EF484279	DQ466851	EF468286	EF471805	EF472820	EF484076			DQ466815	AY307181
Sturnidae	*Aplonis brunneicapillus*	EF486734	EF486734	EF484175		EF468145	EF468283	EF471802	EF472817					
Sturnidae	*Aplonis cantoroides*	EF486735	EF486735	EF484176	EF484277	EF468146	EF468284	EF471803	EF472818.	EF484074				
Sturnidae	*Aplonis cinerascens*	EF486736	EF486736	EF484177	EF484278	EF468147	EF468285	EF471804	EF472819	EF484075				
Sturnidae	*Aplonis grandis*	EF486738	EF486738	EF484179	EF484280	DQ466852	EF468287	EF471806	EF472821	EF484077			DQ466816	DQ466795
Sturnidae	*Aplonis insularis*	EF486739	EF486739	EF484180	EF484281	EF468150	EF468288	EF471807	EF472822	EF484078				
Sturnidae	*Aplonis metallica*	EF486740	EF486740	EF484181	EF484282	EF468151	EF468289		EF472823	EF484079				
Sturnidae	*Aplonis minor*	EF486741	EF486741	EF484182	EF484283	EF468152	EF468290	EF471809	EF472824	EF484080				
Sturnidae	*Aplonis panayensis*	EF486742	EF486742	EF484183	EF484284	DQ466853	EF468291	EF471810	EF472825	EF484081	EU551929	EU551898	DQ466817	AY307182
Sturnidae	*Aplonis pelzelni*	EF486743	EF486743		EF484285	EF468154	EF468292	EF471811	EF472826	EF484082				
Sturnidae	*Aplonis tabuensis*	EF486744	EF486744	EF484185	EF484286	DQ466854	EF468293	EF471812	EF472827	EF484083			DQ466818	DQ466796
Sturnidae	*Basilornis celebensis*	EF486745	EF486745	EF484186	EF486341	EF468156	EF468294	EF471813	EF472828	EF484084				
Sturnidae	*Cinnyricinclus leucogaster*	EF486695	EF486695	EF484136	EF484238	DQ466858	EF468247	EF471766	EF472788	EF484036	EU551930	EU551899	DQ466822	DQ466797

Family	Species	MT-ATP6	MT-ATP8	MT-CO1	MT-CO2	MT-ND2	Fib5	Fib7	RDP1	TGFB2-4	ODC	GAPDH-11	myo	RAG-1
Sturnidae	*Creatophora cinerea*	EF486760	EF486760	EF484201	EF484301	AY329420	EF468308	EF471827	EF472841	EF484098	EU551913	EU551882	DQ466824	AY307189
Sturnidae	*Enodes erythrophris*					EF468227								
Sturnidae	*Fregilupus varius*				EU551999	EU551952								
Sturnidae	*Goodfellowia miranda*	EF486746	EF486746	EF484187	EF484287	EF468157	EF468295	EF471814	EF472829	EF484085				
Sturnidae	*Gracula ptilogenys*					EF468237								
Sturnidae	*Gracula religiosa*	NC_015898	NC_015898	NC_015898	NC_015898	NC_015898	EF468296	EF471815	EF472830	EF484086	EU551931	EU551900	DQ466825	AY307193
Sturnidae	*Gracupica contra*			KC439338	EU552006	EF468175					EU551918	EU551887	EU551864	
Sturnidae	*Gracupica nigricollis*	NC_020423	NC_020423	NC_020423	NC_020423	NC_020423	EF468310	EF471830	EF472842	EF484101	EU551921	EU551890	DQ466844	DQ466809
Sturnidae	*Grafisia torquata*					EF468226								
Sturnidae	*Hartlaubius auratus*	EF486731	EF486731	EF484172	EF484273	DQ466876		EF471799	EF472814	EF484070	EU551935	EU551904	DQ466839	DQ466804
Sturnidae	*Hylopsar cupreocauda*					EF468230								
Sturnidae	*Hylopsar purpureiceps*			JQ175204		EF468214								
Sturnidae	*Lamprotornis acuticaudus*					EF468221								
Sturnidae	*Lamprotornis albicapillus*	EF486730	EF486730	EF484171	EF484272	EF468141	EF468279	EF471798	EF472813	EF484069				
Sturnidae	*Lamprotornis australis*	EF486697	EF486697	EF484138	EF484239	EF468108	EF468249	EF471768	EF472789	EF484038				
Sturnidae	*Lamprotornis bicolor*	EF486732	EF486732	EF484173	EF484274	DQ466879	EF468281	EF471800	EF472815	EF484071			DQ466841	DQ466806
Sturnidae	*Lamprotornis caudatus*	EF486699	EF486699	EF484140	EF484241	DQ466863	EF468251	EF471770	EF472790	EF484040			DQ466826	DQ466799
Sturnidae	*Lamprotornis chalcurus*					EF468240								
Sturnidae	*Lamprotornis chalybaeus*	EF486700	EF486700	EF484141	EF484242	DQ466864	EF468252	EF471773	EF472791	EF484041			DQ466827	DQ466800
Sturnidae	*Lamprotornis chloropterus*					EF468232								
Sturnidae	*Lamprotornis fischeri*	EF486704	EF486704	EF484145	EF484246	EF468115	EF468256	EF471775	EF472794	EF484045				

Family	Species	MT-ATP6	MT-ATP8	MT-CO1	MT-CO2	MT-ND2	Fib5	Fib7	RDP1	TGFB2-4	ODC	GAPDH-11	myo	RAG-1
Sturnidae	*Lamprotornis hildebrandti*	EF486705	EF486705	EF484146	EF484247	EF468116	EF468257	EF471776	EF472795	EF484046				
Sturnidae	*Lamprotornis iris*	EF486707	EF486707	EF484148	EF484249	EF468118	EF468259	EF471778	EF472796	EF484048				
Sturnidae	*Lamprotornis mevesii*	EF486709	EF486709	EF484150	EF484251	EF468120	EF468260	EF471779	EF472797	EF484050				
Sturnidae	*Lamprotornis nitens*	EF486710	EF486710	EF484151	EF484252	AY329425	EF468261	EF471780	EF472798	EF484051				
Sturnidae	*Lamprotornis ornatus*					EF468229								
Sturnidae	*Lamprotornis pulcher*	EF486712	EF486712	EF484153	EF484254	EF468123								
Sturnidae	*Lamprotornis purpureus*	EF486713	EF486713	EF484154	EF484255	EF468124	EF468263	EF471782	EF472799	EF484053				
Sturnidae	*Lamprotornis purpuroptera*	EF486714	EF486714	EF484155	EF484256	EF468125	EF468264	EF471783	EF472800	EF484054	EU551932	EU551901	EU551872	
Sturnidae	*Lamprotornis regius*	EF486716	EF486716	EF484157	EF484258	EF468127	EF468266	EF471785	EF472802	EF484056				
Sturnidae	*Lamprotornis shelleyi*					EF468215								
Sturnidae	*Lamprotornis splendidus*	EF486717	EF486717	EF484158	EF484259	DQ466866	EF468267	EF471786	EF472803	EF484057			DQ466829	AY307194
Sturnidae	*Lamprotornis superbus*	EF486718	EF486718	EF484159	EF484260	EF468129	EF468268	EF471787	EF472804	EF484058				AY307195
Sturnidae	*Lamprotornis unicolor*	EF486720	EF486720	EF484161	EF484262	EF468131								
Sturnidae	*Leucopsar rothschildi*	MN356237	MN356237	MN356237	MN356237	MN356237	EF468311	EF471831	EF472843	EF484102	EU551914	EU551883	EU551861	
Sturnidae	*Mino anais*	EF486749	EF486749	EF484190	EF484290	DQ466868	EF468298	EF471817	EF472831	EF484088			DQ466830	AY307200
Sturnidae	*Mino dumontii*					DQ469047								
Sturnidae	*Mino kreffti*	EF486750	EF486750	EF484191	EF484291	EF468161	EF468299	EF471818	EF472832	EF484089				
Sturnidae	*Neocichla gutturalis*					EF468216								
Sturnidae	*Notopholia corusca*	EF486703	EF486703	EF484144	EF484245	DQ466865	EF468255	EF471774	EF472793	EF484044			DQ466828	DQ466801
Sturnidae	*Onychognathus albirostris*					EF468236								
Sturnidae	*Onychognathus blythii*					EF468245								

Family	Species	MT-ATP6	MT-ATP8	MT-CO1	MT-CO2	MT-ND2	Fib5	Fib7	RDP1	TGFB2-4	ODC	GAPDH-11	myo	RAG-1
Sturnidae	*Onychognathus frater*					EF468246								
Sturnidae	*Onychognathus fulgidus*					EF468224								
Sturnidae	*Onychognathus morio*	EF486721	EF486721	EF484162	EF484263	DQ466869	EF468270	EF471789	EF472805	EF484060			DQ466831	DQ466802
Sturnidae	*Onychognathus nabouroup*					EF468222								
Sturnidae	*Onychognathus salvadorii*	EF486723	EF486723	EF484164	EF484265	EF468134	EF468272	EF471791	EF472806	EF484062				
Sturnidae	*Onychognathus tenuirostris*	EF486724	EF486724	EF484165	EF484266	DQ466870	EF468273	EF471792		EF484063			DQ466832	DQ466803
Sturnidae	*Onychognathus tristramii*	EF486725	EF486725	EF484166	EF484267	EF468136	EF468274	EF471793	EF472808	EF484064				
Sturnidae	*Onychognathus walleri*	EF486726	EF486726	EF484167	EF484268	EF468137	EF468275	EF471794	EF472809	EF484065	EU551933	EU551902	EU551873	
Sturnidae	*Pastor roseus*	EF486768	EF486768	EF484209	EF484309	EF468181	EF468315	EF471835	EF472847	EF484106	EU551924	EU551893	EU551869	
Sturnidae	*Pholia sharpii*	EF486729	EF486729	EF484170	EF484271	DQ466871	EF468278	EF471797	EF472812	EF484068			DQ466833	AY307187
Sturnidae	*Poeoptera kenricki*	EF486727	EF486727	EF484168	EF484269	EF468138	EF468276	EF471795	EF472810	EF484066				
Sturnidae	*Poeoptera lugubris*	EF486728	EF486728	EF484169	EF484270	EF468139	EF468277	EF471796	EF472811	EF484067			DQ466834	AY307207
Sturnidae	*Poeoptera stuhlmanni*					EF468218								
Sturnidae	*Poeoptera femoralis*					EF468217								
Sturnidae	*Rhabdornis inornatus*	EF486774	EF486774	EF484215		DQ466873	EF468321	EF471841	EF472853	EF484112			DQ466836	AY320000
Sturnidae	*Rhabdornis mystacalis*	EF486775	EF486775	EF486342	EF484316	EF468190		EF471842	EF472854	EF484113	EU551934	EU551903	DQ466837	AY307210
Sturnidae	*Sarcops calvus*	EF486752	EF486752	EF484193	EF484293	DQ466875	EF468301	EF471820	EF472834	EF484091			DQ466838	AY307212
Sturnidae	*Saroglossa spilopterus*					EF468220								
Sturnidae	*Scissirostrum dubium*	EF486753	EF486753	EF484194	EF484294	EF468164	EF468302	EF471821	EF472835	EF484092				
Sturnidae	*Speculipastor bicolor*	EF486733	EF486733	EF484174	EF484275	EF468219	EF468282	EF471801	EF472816	EF484072				
Sturnidae	*Spodiopsar cineraceus*	NC_015237	NC_015237	NC_015237	NC_015237	NC_015237	SRR10053857	SRR10053857	SRR10053857	SRR10053857	SRR10053857	SRR10053857	SRR10053857	SRR10053857

Family	Species	MT-ATP6	MT-ATP8	MT-CO1	MT-CO2	MT-ND2	Fib5	Fib7	RDP1	TGFB2-4	ODC	GAPDH-11	myo	RAG-1
Sturnidae	*Spodiopsar sericeus*	NC_014455	NC_014455	NC_014455	NC_014455	NC_014455	SRR10053850	SRR10053850	SRR10053850	SRR10053850	SRR10053850	SRR10053850	SRR10053850	SRR10053850
Sturnidae	*Streptocitta albicollis*	EF486751	EF486751	EF484192	EF484292	EF468162	EF468300	EF471819	EF472833	EF484090				
Sturnidae	*Sturnia erythropygia*				EU552008	EU551960					EU551919	EU551888	EU551865	
Sturnidae	*Sturnia malabarica*	EF486766	EF486766	EF484207	EU552009	EF468178	EF468313	EF471833	EF472845	EF484104	EU551920	EU551889	EU551866	
Sturnidae	*Sturnia pagodarum*	EF486772	EF486772	EF484213	EF484313	EF468187	EF468319	EF471839	EF472851	EF484110	EU551922	EU551891	EU551867	
Sturnidae	*Sturnia sinensis*				EU552019	EF468183					EU551926	EU551895	DQ466845	DQ466810
Sturnidae	*Sturnornis albofrontatus*				EU552002	EU551955					EU551915	EU551884	EU551862	
Sturnidae	*Sturnus unicolor*	EF486770	EF486770	EF484211	EU552022	EU551972	EF468317	EF471837	EF472849	EF484108	EU551928	EU551897	DQ466846	DQ466811
Sturnidae	*Sturnus vulgaris*	NC_029360	NC_029360	NC_029360	NC_029360	NC_029360	EF468318	EF471838	EF472850	EF484109	EU154804	GCF_001447265	GCF_001447265	AY057032
Outgroup	*Ficedula albicollis*	NC_021621	NC_021621	NC_021621	NC_021621	NC_021621	GCF_000247815	GCF_000247815	GCF_000247815	GCF_000247815	GCF_000247815	GCF_000247815	GCF_000247815	GCF_000247815
Outgroup	*Ficedula zanthopygia*	NC_015802	NC_015802	NC_015802	NC_015802	NC_015802					KJ931307		KJ952160	

Table S6. **Species of mitogenomes examined in this study.**

Family	Species	Source/	Accession	Reference
Buphagidae	*Buphagus erythrorynchus*	-	MN356312	Unpublished
Mimidae	*Mimus polyglottos*	SRR8236678	BK010995	This study
Mimidae	*Toxostoma redivivum*	-	MN356247	Unpublished
Sturnidae	*Acridotheres cristatellus*	AHNU-K0041	NC_015613	This study
Sturnidae	*Acridotheres tristis*	AHNU-K0056	NC_015195	This study
Sturnidae	*Gracupica nigricollis*	AHNU-K0200	NC_020423	This study
Sturnidae	*Leucopsar rothschildi*	-	MN356237	Unpublished
Sturnidae	*Spodiopsar cineraceus*	AHNU-K0067	NC_015237	This study
Sturnidae	*Spodiopsar sericeus*	AHNU-K0062	NC_014455	Qian et al. 2013
Sturnidae	*Sturnus vulgaris*	Sv009	NC_029360	Rollins et al. 2016

Table S7. **The ENC and GC3s of PCGs examined in this study.**

Species	ENC	GC3s
Acridotheres cristatellus	39.140	0.520
Acridotheres tristis	39.570	0.526
Buphagus erythrorynchus	42.022	0.528
Gracupica nigricollis	37.195	0.498
Leucopsar rothschildi	38.918	0.513
Mimus polyglottos	38.411	0.518
Spodiopsar cineraceus	38.787	0.517
Spodiopsar sericeus	38.402	0.511
Sturnus vulgaris	38.779	0.518
Toxostoma redivivum	36.881	0.493

Table S8. The best Bayesian evolutionary models of combined dataset.

Data type	Locus	Size (bp)	Model	Model setting	
				Lset nst	rate
Mitochondrial PCG	MT-ATP6	684	GTR+I+G4	6	invgamma
	MT-ATP8	168	GTR+I+G4	6	invgamma
	MT-CO1	1,551	GTR+I+G4	6	invgamma
	MT-CO2	684	GTR+I+G4	6	invgamma
	MT-ND2	1,044	GTR+I+G4	6	invgamma
Nuclear intron	Fib5	553	HKY+G4	2	gamma
	Fib7	894	GTR+G4	6	gamma
	RDP1	1,004	K80+G4	2	gamma
	TGFB2-4	571	K80+G4	2	gamma
	ODC	684	HKY+G4	2	gamma
	GAPDH-11	286	K80	2	equal
	myo	729	K80+G4	2	gamma
	RAG-1	2,872	GTR+I+G4	6	invgamma

Table S9. Determination of substitution saturation of PCGs for combined dataset.

Gene	Codon position	Iss[a]	Iss.cSym[b]	*P*	Iss.cAsym[c]	*P*
MT-ATP6	Codon 1	0.096	0.777	0	0.762	0
	Codon 2	0.026	0.777	0	0.762	0
	Codon 3	0.482	0.777	0	0.762	0
MT-ATP8	Codon 1	0.171	0.899	0	1.021	0
	Codon 2	0.105	0.899	0	1.021	0
	Codon 3	0.318	0.899	0	1.021	0
MT-CO1	Codon 1	0.221	0.797	0	0.763	0
	Codon 2	0.276	0.797	0	0.763	0
	Codon 3	0.669	0.797	0	0.763	0
MT-CO2	Codon 1	0.053	0.777	0	0.762	0
	Codon 2	0.031	0.777	0	0.762	0
	Codon 3	0.413	0.777	0	0.762	0
MT-ND2	Codon 1	0.147	0.784	0	0.756	0
	Codon 2	0.071	0.784	0	0.756	0
	Codon 3	0.073	0.784	0	0.756	0

Note: a. Iss: index of substitution saturation; b. Iss.cSym: critical index of substitution saturation (assuming a symmetrical topology); c. Iss.cAsym: critical index of substitution saturation (assuming an asymmetrical topology).
